# The feasibility of a theory-based self-regulation intervention in schools to increase older adolescents' leisure time physical activity behavior

**DOI:** 10.3934/publichealth.2018.4.421

**Published:** 2018-11-09

**Authors:** James Matthews, Aidan P Moran, Amanda M Hall

**Affiliations:** 1 School of Public Health, Physiotherapy, and Sports Science, University College Dublin, Belfield, Dublin, Ireland; 2 School of Psychology, University College Dublin, Belfield, Dublin, Ireland; 3 Faculty of Medicine, Memorial University, Newfoundland, Canada

**Keywords:** behavior change, leisure time physical activity, youth, school-based intervention

## Abstract

The purpose of this study was to test the feasibility of a theory-based self-regulation intervention to increase older adolescents' leisure time physical activity (LTPA) behavior. Forty-nine adolescents (M = 15.78 years; SD = 0.52; 31% female) from two schools agreed to participate. Participants were randomly assigned to the experimental or control arm at the school level. The experimental group (n = 24) underwent a brief self-regulation intervention for six weeks. The control group (n = 25) continued with standard classes. Intervention fidelity data was collected to assess feasibility. Outcome measures included self-reported LTPA behavior and self-regulation technique use. Intervention sessions were delivered as intended, participant attendance was high and compliance with intervention content was acceptable. The experimental group reported higher levels of LTPA behavior eight weeks post-intervention and increased use of self-regulation techniques. A brief theory-based self-regulation intervention delivered in a school-setting appears feasible and may increase LTPA behavior and self-regulation in adolescents.

## 1. Introduction

Regular physical activity is well documented to provide a range of physical and mental health benefits to adolescents [1],[2]. However, despite these benefits, rates of physical activity decline substantially in the teenage years, with only a small proportion of teenagers in developed countries meeting the recommended guidelines of 60 minutes of moderate to vigorous physical activity per day [3]. Low rates of physical activity during childhood and adolescents are a marker for physical inactivity in adulthood [4]. Thus, there is an urgent need for effective interventions to promote regular physical activity during adolescence.

### 1.1. School-based physical activity interventions

Schools have been recognized as having an important role in promoting adolescent physical activity behavior, with considerable evidence supporting their positive impact [5]–[8]. However, many of the school-based trials conducted have focused on children or young adolescent populations (i.e., 10–14 years of age) [9]–[11]. As a result, there is a gap in our understanding of the effects of school-based interventions targeting older adolescents (i.e., 15–19 years of age) [12]. Furthermore, while these school-based programs appear effective at increasing activity during school hours, much less is known about how effective school-based interventions may be for leisure time physical activity (LTPA) rather than school-based activity [7],[13]. Leisure time physical activity is especially important for older adolescents as they begin to transition out of a structured school environment and increasingly must regulate their own physical activity behavior. Therefore, developing interventions that specifically promote LTPA provides an opportunity to positively influence physical activity behavior in the long term.

### 1.2. Promoting LTPA in adolescents through self-regulation

Self-regulation has been recognized as important for the initiation and maintenance of physical activity [14] and is particularly important when physical activity becomes increasingly under the control of the individual during the transition to adolescence. Self-regulation, defined as personal control of goal directed behavior, is believed to function through three sub processes: (i) self-observation, (ii) self-judgement and (iii) self-reaction [15]. While there is considerable evidence supporting the role of self-regulatory processes in increasing physical activity in an adult population [16], there has been less investigation among adolescents [17]. The majority of work in the adolescent population has been restricted to cross-sectional studies. These studies lack the ability to draw conclusions regarding the effectiveness of self-regulation interventions on physical activity. Therefore, there is a need for greater experimental research in this area [18]. Indeed, of the few intervention studies exploring self-regulation and adolescent physical activity, many have been hindered by the common issue of either not measuring or reporting the actual techniques that underlie the construct of self-regulation [19]. Thus, the particular techniques or underlying processes of self-regulation that are most effective in facilitating physical activity behavior in an adolescent population are not adequately understood. Consequently, there is an increased need for longitudinal and further experimental research exploring self-regulation and adolescent physical activity behavior [20].

### 1.3. A theoretical model of self-regulation

**Figure 1.**
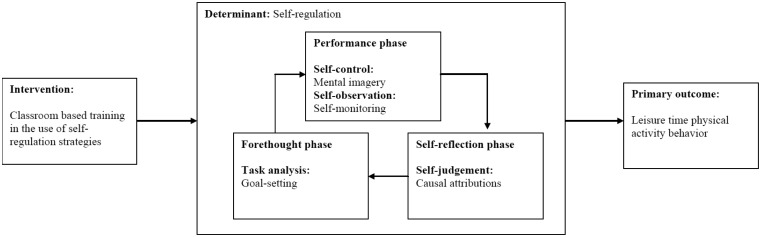
Process model of the intervention which incorporates the cyclical phases and related techniques of self-regulation.

The inclusion of theory within health behavior change interventions has been endorsed by the Medical Research Council [21].However, despite the purported benefits of applying theory to interventions, the evidence for effectiveness is mixed. A scoping review of behavior change theories identified over 80 theories in health related research, however, four theories (Transtheoretical Model, Social Cognitive Theory, Theory of Planned Behavior, Information-Motivation-Behavioral-Skills Model) accounted for 63% of the articles included within this review, leading to a call for greater application and testing of different theories with health related behaviors, such as physical activity [22]. Zimmerman‘s model of self-regulation [23] is one such theory and provided the theoretical framework for the current study. This model has been successfully applied to education [24] and sport [25] but has yet to be applied to the physical activity domain. According to the tenets of this model, self-regulation can be described as self-generated thoughts, feelings and behaviors that are planned and cyclically adapted based on behavioral feedback to attain self-set goals [26]. This process can be represented by three cyclical phases, (i) forethought, (ii) performance, and (iii) self-reflection (see Figure 1). The “forethought” phase refers to processes that precede efforts to engage in the desired behavior and include goal-setting. The “performance” phase involves processes that improve the quality of behavior, such as self-control and self-observation. These processes include techniques such as mental imagery and self-monitoring. Finally, the “self-reflection” phase involves processes that occur after a behavioral effort, that influence a person's reaction to that experience for example, self-judgement and self-reaction [27]. Here, individuals evaluate their performance of the behavior, and attribute the outcome to a perceived cause. The model posits that the engagement in these phases of self-regulation enhances an individual's performance of the behavior and their motivational beliefs about future behavior.

### 1.4. Building on previous research to develop a LTPA intervention for older adolescents

Previous reviews of school-based interventions as a means to increase youth physical activity tend to be broadly supportive [8] with some inconsistency in magnitude of effect which may be due in part to the heterogeneity of the interventions and how physical activity is measured. However, a recent systematic review of school-based interventions highlighted how few studies have assessed intervention fidelity, adaptation or intervention integrity which limits understanding of whether the observed effects are actually due to the intervention itself [28]. The review authors recommended a more detailed understanding of implementation issues in the development of interventions to promote successful scale-up and adoption. Not surprisingly, a lack of thorough development and testing has been observed in many complex interventions aiming to promote behavior change across various health contexts. As such, the Medical Research Council guidelines highlight the importance of research that focuses on using and testing theory in feasibility studies to identify issues with delivery and implementation of the intervention, participant compliance and acceptability and which explores the potential for credibility [21]. Therefore, what is needed in the field of school-based physical activity intervention research, is more comprehensive studies that use and test theory and can provide accurate information on recruitment, data collection methods, intervention implementation and acceptability, so the feasibility of these programs can be fully assessed prior to scaling up to a larger trial.

### 1.5. Study aim

The aim of this study was to test the feasibility of a theory-based self-regulation intervention delivered in a school setting to increase older adolescents' LTPA behavior. Specifically, this included assessment of study recruitment rates, implementation of the intervention, its acceptability and finally, the feasibility of an objective measure of physical activity. A secondary objective was to evaluate the contributing assumption of the underlying theoretical model of self-regulation. Specifically, we hypothesised that training in self-regulation techniques would lead to an increase in the use of self-regulation techniques by the experimental group as compared to the control group and that there would be an increase in LTPA behavior by the experimental group as compared to the control group (see Figure 1).

## 2. Materials and methods

### 2.1. Setting, randomisation, and ethics

Two mixed-sex schools with an inter-denominational ethos were invited and agreed to participate in this study. The schools were matched in that both were located in Dublin, Ireland, were classified as “large” schools (>800 students) by the government's Department of Education and Skills and had a similar ratio of male to female students. Neither school was classified as disadvantaged by the Department, as measured by student retention rates and by the number of student families who had access to health services free of charge. This access is based on the family income being below a certain figure. Both schools had also received positive inspection reports from the Department of Education and Skills school review process and had similar practices with respect to physical education. The schools were randomly assigned to one of the study conditions (i.e., intervention or control group) using a computer generated randomization process. The study received ethical approval from the Human Research Ethics Committee of a University and also approval from the boards of management at the participating schools.

### 2.2. Participants

Within each school, tenth grade students were approached to take part. Parents or guardians provided informed written consent for their child to participate and students provided their assent. All students who provided informed consent and expressed an interest in participating were invited to take part. There were no restrictions on how active the students were.

### 2.3. Intervention description

The intervention is described in line with the TIDieR guidelines for intervention development and replication [29] and includes content, delivery, dose and fidelity information. The TIDieR checklist is reported in Table 1 of the supplemental file.

#### 2.3.1. Why: the rationale and theoretical underpinning of the intervention

The intervention was based on Zimmerman's model of self-regulation [23] and techniques were chosen from across the three phases of the model. Goal-setting was chosen from the forethought phase, self-monitoring and mental imagery from the performance phase and strategic (causal) attributions from the self-reflection phase. Recent research highlights the relevance of each technique within the physical activity domain. Specifically, goal-setting has been identified as an effective strategy to promote physical activity behavior in youth populations [30]. Self-monitoring is believed to be fundamental to the self-regulatory process in health related behaviors such as physical activity [16]. Mental imagery has been used as a technique to promote increased physical activity in adolescent populations [31]. Finally, attribution training focused on how increasing a person's feeling of control can increase their physical activity behavior [32].

#### 2.3.2. Who, what, where and how

The experimental group received a standardized training program based on the self-regulation techniques described in the previous paragraph. It was delivered during the spring term and all sessions were conducted on school premises during standard school hours. In line with a number of school-based physical activity interventions [33], it was an education based program and consisted of weekly classroom sessions for 30 minutes over a six week period, where students were prompted to practice the particular techniques with their LTPA in their own time [12]. (Students' continued with their standard physical education class). The intervention program engaged participants in a range of learning activities to develop these self-regulatory techniques and how they could be applied to their LTPA. To support this, the sessions were developed from a social constructivist viewpoint [34]. Specifically, three social constructivist principles were applied. First, the intervention deliverer acted as a facilitator promoting peer interaction and collaboration and encouraging the students to be active participants in the sessions. Second, participants were encouraged to share their prior experiences and their current understanding of these techniques to help construct knowledge. Finally, to create authentic and meaningful learning experiences, the participants applied the self-regulatory techniques to their own chosen LTPA behavior and were then prompted to try out these techniques in their lives, outside of the classroom (i.e., with their LTPA behavior) [35],[36]. To support these social constuctivist principles, didactic lectures using PowerPoint were kept to a minimum; group discussions, short group-based case studies/exercises, and personal reflection activities were core components of every session. Further details of the intervention content and training program can be found in Table 1.

In this feasibility study, the intervention was primarily delivered by the lead author who was experienced leading training programs and working with an adolescent cohort. A teacher from the school attended the intervention sessions and acted as a support in facilitating group discussion and group exercises. This teacher was sent the outline of each session in advance and discussed the session with the lead author prior to delivery to ensure understanding of their role during the particular session. Prior to the delivery of the intervention, the program content and structure was reviewed by two psychologists and two school teachers. The psychologists had published research in related fields and had experience working with adolescents. Both teachers had over five years' experience working with older adolescents. This review panel deemed the content and structure to be appropriate to the study aims and population.

#### 2.3.3. How well (Intervention fidelity)

Intervention delivery was assessed using a self-report checklist completed by the lead author to confirm if the relevant intervention session materials had been used and session content and structure had been followed. Intervention receipt was assessed by participant attendance at each session. Intervention enactment was examined post-intervention with a 5-item participant questionnaire assessing use of self-regulatory techniques with respect to their LTPA behavior.

### 2.4. Control group

Participants were invited to complete the study measures at baseline and follow up. It was explained that the study was exploring the typical LTPA behavior of adolescents and to do so, participants would complete a number of measures twice. No further information on the underlying premise of the study was provided. Participants in the control group had six classroom sessions that were not related to physical activity or self-regulation and they continued with their standard physical education classes for the six week period. This also took place during the same spring term as the intervention.

### 2.5. Outcome assessment

The study took place during standard school hours. Measurements were taken at baseline and at 14 weeks (eight weeks post-intervention) for both the intervention and control participants. LTPA behavior was measured with a subjective assessment tool as the main measure and an objective tool as a supplementary measure. Self-regulation technique use was also measured.

### 2.6. Measures

The primary and secondary outcome measures are described below. The self-regulation measures are briefly listed below and a more detailed description, including the psychometric properties of each measure are provided in Table 2.

#### 2.6.1. Leisure time physical activity

The Godin Leisure-Time Physical Activity questionnaire (GLTPAQ) [37] was used as the primary assessment tool to measure LTPA behavior and has been used with adolescent populations [38]. The measure has been shown to have acceptable reliability (i.e., test–retest over 2-week interval correlation coefficient = .81) [39] and validity (age–sex adjusted correlation between GLTPAQ score and accelerometer METs = .32) [40]. This scale assesses the frequency of weekly leisure time physical activity at mild, moderate and vigorous intensities. Participants were provided with examples of each type of activity and were then asked to state how often they engage in each type for at least 30 minutes at a time in a typical week. Physical activity that occurred outside of their leisure time was excluded (e.g., activity as part of physical education lessons). Only the moderate and vigorous categories were included in the analysis for the present study, this was to align with the public health recommendations for physical activity. For the GLTEQ, a composite metabolic equivalent total value (MET) is calculated and reported. This is done by multiplying the frequencies of moderate and vigorous activities by nine and five respectively and then summing the products. This composite MET value has been used with adolescent populations [41],[42] and also as a measure of change over time in physical activity behavior [43].

The Yamax SW701 Digiwalker pedometer was used as a secondary measure of LTPA as an attempt to supplement the self-report questionnaire with additional objective data. Pedometers were provided to all participants at baseline after questionnaire completion. Participants were to wear the pedometer for four consecutive days (including at least one weekend day) and were provided with a demonstration and written instructions as to how correctly wear the pedometers. The process for recording data reported by Lubans et al. was followed [44]. The pedometers were not sealed. To attempt to offset reactivity and tampering issues participants were encouraged not to alter their behavior based on their daily step count across these four days of data collection. Four days of usable data were required for calculation. Days were excluded if the pedometer was removed for greater than one hour per day or any day with a step count of less than 1000 steps or greater than 30,000 steps. The total number of steps was divided by the number of days worn.

#### 2.6.2. Self-regulation techniques

- *Goal-setting*. This technique was measured using an amended goal-setting subscale from the Test of Performance Strategies (TOPS) [45].
- *Self-monitoring*. This was assessed using a subscale from a self-regulation measure [46].
- *Mental imagery.* This was measured using subscales from the revised exercise imagery questionnaire which focused on the motivational elements of mental imagery (EII–R) [47].
- *Causal attribution.* The importance of controllability for the execution of behavior has been emphasized. Therefore, the controllability subscales from the Revised Causal Dimension Scale (CDS-II) [48] were used in this study. (See Table 2 for further information on these measures).

### 2.7. Statistical analyses

Data was analysed using SPSS (version 20) and all data was checked for accuracy.The effects of the intervention on the primary and second outcomes were calculated using linear mixed models that incorporated terms for group allocation, time and a group allocation by time interaction with a random-effects term for student to account for within-participant repeated measures. An intention to treat analysis was followed. Missing data was dealt with by multiple imputation [49]. Due to the small number of cases in this feasibility study that could affect power to detect differences in the primary or secondary outcomes, effect sizes (i.e., Cohen's d) were also generated to explore the trends and potential effects of the intervention from baseline to follow-up. This particular measurement approach suggests 0.20, 0.50 and 0.80 represent small, medium and large effect sizes respectively [50].

## 3. Results

### 3.1. Participants

**Figure 2.**
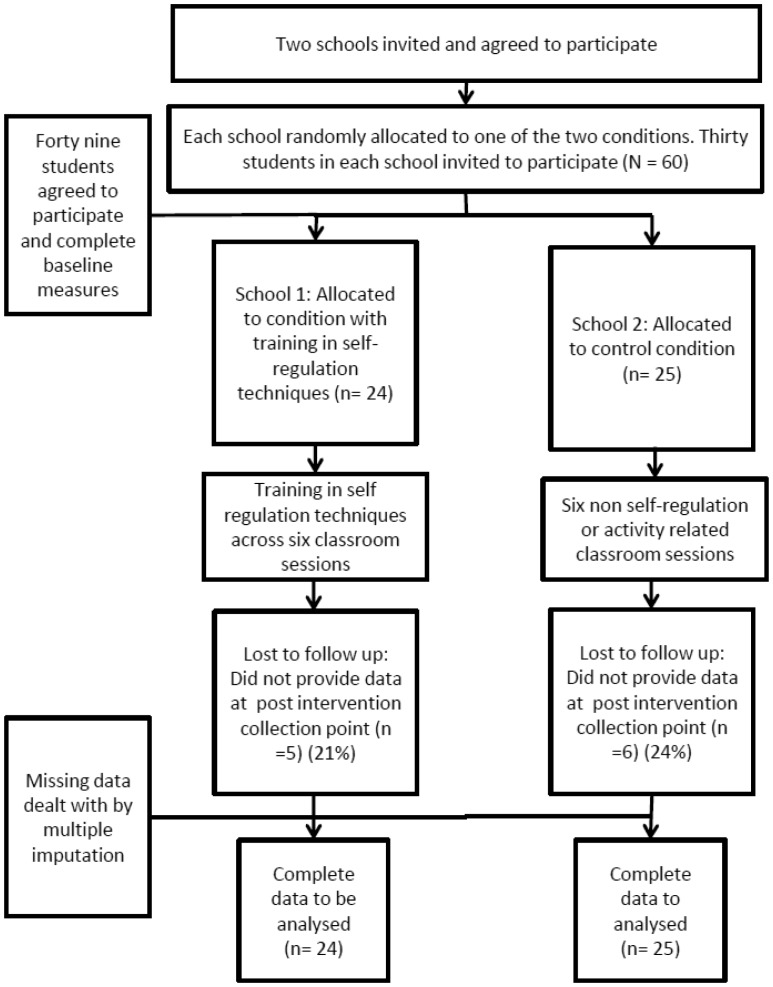
Participant flow diagram

A total of 60 students were invited to participate (30 students from each school). Forty nine students agreed to participant in the study; 24 in the experimental group and 25 in control group (see Figure 2). The mean age of the participants was 15.78 (SD = 0.52), with 31% of the sample consisting of female adolescents. The mean LTPA behavior reported by participants equated to 49.12 METs (SD = 28.26) per week. Of the 49 participants, there were no drop-outs and all started the intervention.

### 3.2. Intervention fidelity

#### 3.2.1. Session materials, structure and content

In general, all sessions were delivered as intended with respect to the structure and content of the session, and all the relevant materials were used. However, one element of Session 1 (i.e., short case study) was not completed as intended due to time constraints. This case study was moved to Session 2 for completion.

#### 3.2.2. Session attendance

For the experimental group, there was high attendance at the intervention sessions. Average attendance across the six sessions was 87%, with a high of 96% at Sessions 1 and 6, and a low of 79% at Sessions 2, 3 and 4. Typical reasons for non-attendance were absence from school or participation at alternative curricular activities.

#### 3.2.3. Reported use of self-regulation activities

At eight weeks post-intervention, self-reported use of the self-regulation techniques was as follows:

- *Goal-setting*. 90% of participants in the experimental group reported setting and planning a physical activity related goal.
- *Self-monitoring*. 53% also reported physically recording their progress towards their goal on a regular basis.
- *Mental imagery*. 84% of participants said they utilised mental imagery with respect to their physical activity goal and 47% reported actively using the imagery paragraph they developed in Session 4.
- *Causal attribution*. 79% reported reorienting their physical activity attributions to focus on personally controllable factors such as effort or strategy.

### 3.3. Outcome measures

Table 3 reports the changes in primary and secondary outcomes. Standardized effect sizes are reported to show effects and trends. A brief description is also provided below.

#### 3.3.1. Leisure time physical activity

Participants in the experimental group reported improvements in levels of moderate to vigorous LTPA behavior from baseline to 14 weeks (eight weeks post-intervention) behavior, with a small within group positive effect reported.

#### 3.3.2. Pedometer

We were unable to calculate this outcome due to large amounts of missing data at both baseline and follow-up assessments. While, most participants reported using the pedometer (baseline: 83%, follow-up: 59%), only eight (16%) participants provided data at both time-points that met the criteria for calculation. Thus, due to the degree of missing data, further analysis on this outcome was deemed inappropriate.

**Table 1.** Intervention self-regulatory techniques, outcomes and content

Schedule	Self-regulatory techniques	Participant session learning outcomes	Session content
Week 1 & 2	Goal-setting^1^ & Self –monitoring^2^	Demonstrate an understanding and apply the principles of SMART goal-setting and self-monitoring to increase LTPA.	Information provided on physical activity and types of leisure time physical activity. Information provided on the principles of goal-setting and self-monitoring and clear rationale as to their relevance for increasing the frequency of LTPA. Group discussion of participants' prior knowledge of goal-setting and self-monitoring and how and why they could help increase LTPA behavior. Case studies & role plays competed in groups, to practice developing realistic SMART physical activity goals and self-monitoring practices. Team quiz to reinforce the basic principles of goal-setting and self-monitoring. Personal reflection on current LTPA behavior and the development of personalized LTPA frequency related SMART goal/s.
Week 3 & 4	Mental imagery^2^	Demonstrate an understanding of how mental imagery can support an individual's LTPA. Develop and use a brief personalized physical activity related imagery paragraph.	Brief review and discussion of previous sessions and participants' experience utilizing goal –setting and self-monitoring. Information provided on mental imagery and clear rationale as to its relevance to LTPA. Group discussion of participants' prior knowledge of mental imagery and how and why it could help increase their frequency of LTPA. Participants undertake a guided imagery exercise. Information provided as to how to develop an effective imagery paragraph. Activity where participants work collaboratively to develop a brief imagery paragraph to support a fictional teenager to increase the frequency of their LTPA behavior. Team quiz to reinforce the basic principles of mental imagery use. Personal reflection and the development of brief personalized imagery paragraph for use to increase the frequency of LTPA in their own lives.
Week 5 & 6	Strategic attributions^3^	Demonstrate understanding as to how beliefs about physical activity behavior can influence future performance. Utilise adaptive attributions to explain LTPA.	Brief review and discussion of previous sessions and participants' experience utilizing goal setting, self-monitoring and mental imagery. Information provided on what attributions are and clear rationale as to its relevance to LTPA. Group activity exploring participants' differing attributions for physical activity behavior. Case study where participants work collaboratively to practice identifying maladaptive attributions and how to develop more adaptive attributions to link towards physical activity behavior Team quiz to reinforce the principles for adaptive and maladaptive attributions. Personal reflection and recording of prompts to encourage adaptive attributions to future LTPA in their own lives.
Materials	Brief PowerPoint lectures; Group activities, Case studies; Quizzes; Program handbook		

Note: LTPA: Leisure time physical activity; ^1^From the forethought phase of the self-regulation model [23]; ^2^From the performance phase of the self-regulation model; ^3^From the self-reflection phase of the self-regulation model.

**Table 2.** Description of self-regulation measures

Measure	Description of the measure	Scale (if applicable)	Reliability and validity
*Goal-setting:* test of performance strategies (TOPS)	An amended goal-setting subscale from this scale was used, containing four items. For example, “I have specific goals for my physical activity”.	5-point Likert scale ranging from 1 (“Never”) to 5 (“Always”).	The measure has demonstrated adequate validity [45] and the adapted subscale has shown to be reliable [18]. In this study, the internal consistency of the scale was α = 0.88.
*Self-monitoring* self-regulation for physical activity.	A sub-scale from this measure with six items was used. Sample items included, “I keep track in my head of how often I am physically active” and “I write down how often I am physically active”.	5-point Likert scale ranging from 1 (“Never”) to 5 (“Always”).	The reliability of the scale has been demonstrated [46].The internal consistency for the sub-scale in the present study was acceptable at α = 0.70.
Mental imagery: revised exercise imagery questionnaire (EII – R)	Two subscales from this measurement tool were used, each had four items. The self-efficacy subscale (e.g., “I imagine having the confidence to complete my workout”), and the feeling subscale (e.g., “I imagine how I will feel after exercising”).	7-point Likert scale from 1(“Never”) to 7 (“Often”).	The EII-R in general and these particular subscales have been shown to be valid and reliable [47]. In the present study, internal consistency for the self-efficacy subscale was α = 0.75, and for the feeling subscale was α = 0.74.
Causal attribution: revised causal dimension scale (CDS-II)	The three-item personal and external controllability subscales from this measure were used to explore participants' causal attributions.	9-point Likert type scale. For each item, there are specific anchors. For example, for an item on personal controllability, 1 (“Not manageable by you”) to 9 (“Manageable by you”).	The CDS-II has appropriate reliability and acceptable construct validity [48]. In this study, the internal consistencies for the personal controllability and external controllability subscale were α =0.82 and α =0.69 respectively.

**Table 3.** Changes in primary and secondary outcome measures for the experimental and control groups and results of the linear mixed models

	Experimental Group (n= 24)			Control Group (n=25)			Time x Group		Adjusted between group difference
Outcome	Baseline, Mean (95% CI)	14 weeks, Mean (95% CI)	Within Group (Experimental) Effect Size (Cohen's d)	Baseline, Mean (95% CI)	14 weeks, Mean (95% CI)	Within Group (Control) Effect Size (Cohen's d)	F (df)	P	14 weeks, (95% CI)
LTPA-MV MET	54.38 (42.82,65.93)	62.86 (51.30,74.41)	0.29	44.08 (32.76,55.40)	44.82 (33.49,56.14)	-0.02	1.12 (1,47)	0.27	11.82 (-1.86,25.50)
Goal-setting	12.29 (10.44,14.14)	14.08 (12.23,15.93)	0.39	11.96 (10.15,13.77)	11.16 (9.35,12.97)	-0.17	5.65 (1,47)	0.02	2.67 (-0.53,4.81)
Self-monitoring	14.58 (12.58,16.59)	17.96 (15.96,19.96)	0.64	13.56 (11.59,15.53)	14.08 (12.12,16.05)	0.1	5.91 (1,47)	0.02	2.93 (0.53,5.33)
Feeling-based imagery	16.79 (13.99,19.59)	22.33 (19.54,25.13)	0.83	16.08 (13.34,18.82)	17.64 (14.89,20.38)	0.22	3.73 (1,47)	0.06	4.30 (0.43,8.18)
Self-efficacy based imagery	14.46 (12.48,16.44)	16.17 (14.19,18.15)	0.37	15.32 (13.38,17.26)	14.80 (12.86,16.74)	-0.1	2.11 (1,47)	0.15	1.72 (-0.96,4.40)
Causal attribution: external control	13.25 (10.84,15.65)	14.21 (11.80,16.61)	0.15	13.32 (10.96,15.68)	13.40 (11.04,15.76)	0.01	0.17 (1,47)	0.68	0.83 (-2.69,4.35)
Causal attribution: personal control	21.08 (18.54,23.63)	21.54 (18.99 24.09)	0.07	20.12 (17.63,22.61)	18.88 (17.63,21.37)	-0.2	1.45 (1,47)	0.23	1.91 (-0.87,4.69)

Note: LTPA MV MET: Leisure time physical activity–Moderate & vigorous, metabolic equivalent total.

#### 3.3.3. Self-regulatory techniques

For all self-regulatory techniques, the findings trended towards the experimental group at follow-up. In the experimental group, there were small to medium positive within group effects for goal-setting, self-monitoring and self-efficacy based imagery. There was a large positive within group effect observed for feeling based mental imagery.

## 4. Discussion and conclusions

The study aim was to test the feasibility of a theory-based self-regulation intervention to increase older adolescents' LTPA behavior, with a secondary objective to evaluate the underlying theoretical model of self-regulation. There were high recruitment rates in this study, and it appears the intervention can be delivered as intended, was received and enacted by most participants, and thus is broadly feasible within this setting. However, it must be noted that the inclusion of an objective measure of physical activity for data collection purposes was unsuccessful. The intervention may also be somewhat efficacious, as the trends highlight an increase in the use of theory-based self-regulation techniques and an increase in moderate to vigorous LTPA in the experimental group.

The importance of intervention implementation in school-based interventions has been highlighted in recent times [28]. In line with these recommendations, data was collected in the present study with the aim of providing initial evidence of the feasibility of offering this type of intervention in school systems. Data from the session checklists, participant attendance and self-regulatory technique compliance records provided information that the intervention does indeed appear to be broadly feasible. Specifically, the sessions were delivered as intended from a structure and content perspective with one small deviation from the protocol, thus highlighting adequate fidelity. It is acknowledged that a self-report checklist is not the most accurate method for assessing fidelity. However, this method may still have a place in ensuring fidelity to an intervention protocol for example, by acting as a memory aid for intervention providers [51]. With respect to receipt, there was high attendance at most intervention sessions. This followed a successful recruitment phase where 82% of students who were approached to take part in the study agreed to participate. This high recruitment rate was facilitated by the research team establishing good relationships with the school principals and teachers, and that the intervention was likely novel as compared to students' traditional classes. For intervention enactment, many adolescents reported using the techniques outside the intervention sessions with their LTPA behavior as intended, suggesting these techniques are somewhat acceptable, and realistic to implement with this age group. However, it must be noted that the reported use of self-monitoring and the self-developed imagery script were lower than other techniques. This may suggest that the training in these specific techniques was less effective or using these techniques as promoted in the intervention may be less acceptable to participants. In addition, we recognise that the compliance measure was somewhat limited in that participants simply reported yes or no as to whether they had used the techniques since the intervention. A more detailed measure or a qualitative methodology may be more appropriate to utilise in any future work [52].

In terms of the adaptability of the intervention, there are some modifications to the intervention content that need to be considered. First, whether the self-regulation techniques selected from the model are the most effective for use with this population. The introduction of problem solving as an additional self-regulatory technique may further augment the goal-setting and monitoring components of the intervention [14], while the inclusion of mindfulness may also enhance the intervention by strengthening emotional self-regulation [53]. Second, there was limited use or promotion of technology within the intervention as it was designed. The inclusion of e-learning programs or mobile applications to support the intervention may further enhance the acceptability and ultimately the efficacy of the program with this technology literate population group [54]. For example, the development of e-content so that participants can complete missed sessions in their own time or the use of mobile applications to support self-monitoring of LTPA [55] or promote the use of mental imagery scripts [56]. Indeed, there have been a number of promising e-learning programs targeting adolescents' physical activity behavior within schools. These e-learning programs can target students directly for example, through a gaming intervention [57], or indirectly for example, through teacher professional development to enhance the delivery of physical education [58]. Finally, the intervention was delivered by the lead author to assess feasibility. The next step in this process would be a pilot efficacy trial [21] set in the wider school system. This would require the adaption of the program in that teachers would need to be trained to deliver the intervention.

There is an increased call for the use and testing of theory to underpin health behavior interventions [22]. Consequently, the secondary objective of the study was to test Zimmerman's model of self-regulation [23] in this domain. This study provides preliminary support for the use of techniques from this three phase model of self-regulation to improve older adolescents' LTPA behavior. More specifically, in line with the proposed model, the intervention appeared to have a larger effect on the process variables than it did on LTPA. A fully powered efficacy trial could explore this more thoroughly using mediation analysis.

This increase in moderate to vigorous LTPA as measured by METs (i.e., 8.48 METs) from baseline to post intervention in the experimental group equates to nearly one additional session of vigorous intensity activity per week as per the GLTEQ classification system [37]. At an individual level, when considering the public health recommendations for this cohort, this may appear a modest increase however, this small change in behavior could have a considerable effect if replicated across a population group. Furthermore, this increase in LTPA is also encouraging when viewed in light of the dose and type of intervention. For example, adolescents in the experimental group only received 30 minutes per week of classroom-based training in the use of self-regulation techniques for six weeks and were simply prompted to try to increase their LTPA behavior. Thus, training older adolescents in theory-based self-regulation techniques might be a simple yet effective way to augment traditional physical education in schools and support this cohort to be more physically active in their leisure time [59]. It is possible that a more intense or sustained intervention with the addition of an actual physical component to the intervention could have a greater impact on the adolescents' LTPA behavior. However, interestingly, a recent review by Hynynen et al. [12] suggested that the inclusion of behavioral practice within school-based intervention sessions targeting older adolescents was more typical of non-effective interventions whereas, simply prompting behavioral practice / rehearsal outside of the intervention may be a more effective approach.

The major strengths of this study relate to the attention given to intervention development and reporting, and outcome and process assessment. Firstly, the intervention was theoretically based and includes an in-depth process model which describes how the intervention is proposed to work; in this way it outlines the particular self-regulation techniques that should be assessed. The self-regulation techniques were measured and reported individually rather than simply reporting under the global term of self-regulation, which was a criticism of previous research [18]. These features in combination enable better understanding as to the underlying processes of the intervention and which techniques might be most important for this cohort [60]. Secondly, the intervention components were described in a manner to support transparency and replication through the use of the TIDieR guidelines [29].

There were also some limitations to this study that should be recognized. First, only two schools were included in this feasibility study. While attempts were taken to ensure they were similar and allocation to conditions was done randomly, future studies should ensure a greater number of schools are included as this will allow the effect of the school to be included as a random effect in the analysis. Two, adolescent LTPA was only assessed using a self-report measure. The study did attempt to objectively measure participants' physical activity using pedometers. Unfortunately, this was not successful as insufficient usable data was collected. Thus, while pedometers may be a viable tool to measure adolescents' physical activity, further research is required to compare different monitoring protocols for pedometer usage with adolescents [44]. There is also a requirement to consider other forms of objective measurement with this cohort [61]. Third, self-report checklists were used to assess the fidelity of intervention delivery. This method of fidelity is less robust than other methods such as independently rated audio or video recordings. Any future research should ensure to apply these alternative fidelity assessment methods [62]. Finally, only students who expressed an interest in participating in the study and had parental consent were allowed to take part, potentially introducing limits on generalizability.

In conclusion, this study provides some preliminary evidence of the feasibility of a theory-based intervention delivered in a school setting training adolescents in the use of self-regulatory techniques to promote LTPA behavior. These findings may inform the design, development and implementation of a revised pilot efficacy study to more adequately test this proposition and assess its scalability to the wider school system. Notwithstanding the requirement for further testing, teachers and sports coaches could be encouraged to consider formally introducing self-regulatory techniques to help older adolescents regulate and engage in LTPA behavior at this important stage of life.
