# Supplementary material for: The feasibility of a theory-based self-regulation intervention in schools to increase older adolescents' leisure time physical activity behavior
Source: AIMS Public Health. 2018 Nov 9;5(4):421–39. doi: 10.3934/publichealth.2018.4.421 (PMC6322992; doi:10.3934/publichealth.2018.4.421)
Supplement: Supplementary file 1 [file publichealth-05-04-421-s001.pdf]

Public Health

AIMS Public Health, 5(4): 421–439.
DOI: 10.3934/publichealth.2018.4.421
Received: 27 June 2018
Accepted: 16 October 2018
Published: 09 November 2018

http://www.aimspress.com/journal/aimsph

Research article

The feasibility of a theory-based self-regulation intervention in schools to increase older adolescents' leisure time physical activity behavior

James Matthews[1,]*, Aidan P Moran[2] and Amanda M Hall[3]

[1] School of Public Health, Physiotherapy, and Sports Science, University College Dublin, Belfield, Dublin, Ireland
[2] School of Psychology, University College Dublin, Belfield, Dublin, Ireland
[3] Faculty of Medicine, Memorial University, Newfoundland, Canada

* Correspondence: Email: james.matthews@ucd.ie; Tel: +35317163449.

Supplemental Table 1. Checklist for adherence to the TIDiER guidelines

Item number	Item	Where located in the manuscript
	**Brief Name:**	
1	Provide the name or phrase that describes the intervention	Page 423, Line 108
	**Why:**	
2	Describe any rationale, theory, or goal of the elements essential to the intervention	Page 424, Line 140
	**What:**	
3	Materials: Describe any physical or informational materials used in the intervention, including those provided to participants or used in intervention delivery or in training of intervention providers. Provide information on where the materials can be accessed (such as online appendix, URL)	Page 425, Line 151–168 & Table 1
4	Procedures: Describe each of the procedures, activities, and/or processes used in the intervention, including any enabling or support activities	
	**Who provided:**	
5	For each category of intervention provider (such as psychologist, nursing assistant), describe their expertise, background, and any specific training given	Page 425, Line 170–178

Continued on next page

Item number	Item	Where located in the manuscript
	**How:**	
6	Describe the modes of delivery (such as face to face or by some other mechanism, such as internet or telephone) of the intervention and whether it was provided individually or in a group	Page 425, Line 152
	**Where:**	
7	Describe the type(s) of location(s) where the intervention occurred, including any necessary infrastructure or relevant features	Page 425, Line 153
	**When and How Much:**	
8	Describe the number of times the intervention was delivered and over what period of time including the number of sessions, their schedule, and their duration, intensity, or dose	Page 425, Line 154
	**Tailoring:**	
9	If the intervention was planned to be personalised, titrated or adapted, then describe what, why, when, and how	N/A
	**Modifications:**	
10	If the intervention was modified during the course of the study, describe the changes (what, why, when, and how)	Page 427, Line 275
	**How well:**	
11	Planned: If intervention adherence or fidelity was assessed, describe how and by whom, and if any strategies were used to maintain or improve fidelity, describe them	Page 425, Line 180–184
12	Actual: If intervention adherence or fidelity was assessed, describe the extent to which the intervention was delivered as Planned	Page 427 & Page 428, Line 256

AIMS Press

© 2018 the Author(s), licensee AIMS Press. This is an open access article distributed under the terms of the Creative Commons Attribution License (http://creativecommons.org/licenses/by/4.0)
